# The Merritt H. Cash Prize Essay

**Published:** 1890-04

**Authors:** 


### THE MERRITT H. CASH PRIZE ESSAY.

Albany, N. Y., March 1, 1890.

*Editors Buffalo Medical and Surgical Journal:*

For the information of all concerned, I desire to state that the Medical Society of the State of New York offers a prize of $100, payable from the Merritt H. Cash Prize Fund, for the best original essay on any medical or surgical subject.

The conditions are: That the competitors shall reside in the State of New York; that the essays shall be either printed or type-written; that each essay shall be designated by a motto on the title page; that a corresponding motto, together with the name of the writer, shall be inclosed in a sealed envelope and attached to the essay; and that all essays shall be sent to the Chairman of the Committee on Prize Essays, Dr. George F. Shrady, New York, prior to January 1, 1891.

F. C. CURTIS,

*Secretary.*

Other members of the Committee are Drs. Daniel Lewis, New York, and Eugene Beach, Gloversville.

